# The impact of the route to diagnosis in nephroblastoma

**DOI:** 10.1002/cam4.7226

**Published:** 2024-05-24

**Authors:** Marvin Mergen, Nils Welter, Rhoikos Furtwängler, Patrick Melchior, Christian Vokuhl, Manfred Gessler, Clemens‐Magnus Meier, Leo Kager, Jens‐Peter Schenk, Norbert Graf

**Affiliations:** ^1^ Department of Pediatric Oncology and Hematology Saarland University Medical Center Homburg Germany; ^2^ Divison of Paediatric Hematology and Oncology, Department of Paediatrics, Inselspital Bern University Hospital, University of Bern Bern Switzerland; ^3^ Department of Radiation Oncology Saarland University Medical Center Homburg Germany; ^4^ Section of Pediatric Pathology, Department of Pathology University Hospital Bonn Bonn Germany; ^5^ Developmental Biochemistry and Comprehensive Cancer Center Mainfranken, Theodor‐Boveri‐Institute/Biocenter University of Würzburg Würzburg Germany; ^6^ Department of General Surgery, Visceral, Vascular and Pediatric Surgery Saarland University Medical Center Homburg Germany; ^7^ Department of Pediatrics St. Anna Children's Hospital, Medical University Vienna Vienna Austria; ^8^ St. Anna Children's Cancer Research Institute Vienna Austria; ^9^ Department of Diagnostic and Interventional Radiology, Division of Pediatric Radiology University Hospital Heidelberg Heidelberg Germany

**Keywords:** nephroblastoma, outcome, patient and tumor characteristics, route to diagnosis

## Abstract

**Introduction:**

Wilms tumor (WT) is the most common childhood kidney cancer. It is a rapid growing embryonal tumor in young children and can be diagnosed with and without tumor related symptoms.

**Methods:**

We retrospectively analyzed the route to diagnosis of WT treated prospectively according to the SIOP 93‐01/GPOH and 2001/GPOH in Germany between 1993 and 2022. Four routes were defined: diagnosis due to tumor‐related symptoms, incidental diagnosis during another disease, diagnosis by preventive examinations, and diagnosis within a surveillance program. For these groups we compared clinical and tumor characteristics and outcome.

**Results:**

Of 2549 patients with WT 1822 (71.5%) were diagnosed by tumor‐related symptoms, 472 (18.5%) incidentally, 213 (8.4%) by preventive medical examinations, and 42 (1.6%) by surveillance. Age, general health status, tumor volume, and local and overall stage varied significantly between these groups. The youngest patients were those diagnosed by preventive medical examination (mean: 1.70 years). These patients also showed the best general health status. Tumor volume at diagnosis (549 mL) and after preoperative chemotherapy (255 mL) was significantly higher for children with tumor‐related symptoms. The highest percentage of local stage I (78.6%) and the lowest percentage of metastatic disease (4.8%) was found in the surveillance group. The outcome of patients was not significantly different, with up to 19.0% relapses in the surveillance group and 3.0% deaths in the group with tumor‐related symptoms.

**Conclusion:**

The route to diagnosis of WT correlates with age, general health status, tumor volume, and stage distribution, but does not impact the outcome of patients. Nonetheless, diagnosis without tumor related symptoms results in lower treatment burden and thus improved quality of life.

## 1 INTRODUCTION

Although nephroblastoma or Wilms tumor (WT) is rare, it is the most common childhood kidney tumor and can even seldomly arise in extra‐renal sites without any association to the kidney.^1^ For more than 50 years this tumor has been treated in prospective multicenter and randomized trials by the International Society of Pediatric Oncology (SIOP), the Childrens' Oncology Group (COG), and others.^2^, ^3^ Outcome has improved over time with a long‐term survival of 90% today.^4^, ^5^ The tumor occurs mainly in young children between 3 and 4 years. In more than 10% of children with WT a cancer predisposition syndrome (CPS) can be diagnosed^6^, ^7^ that may lead to an earlier diagnosis if regular abdominal ultrasounds are performed. As tumor size grows over time and correlates with outcome, early diagnosis should be beneficial.

In this retrospective study we analyzed the route to diagnosis of WT in a large cohort of patients from Germany over a 29‐year period to answer the question, if the outcome of WT‐patients depends on the route to diagnosis.

## 2 PATIENTS AND METHODS

### 2.1 Study design and population

Data of patients with kidney tumors prospectively enrolled into two consecutive studies and trials performed by the German Society of Pediatric Oncology and Hematology (GPOH) (SIOP‐93‐01/GPOH and SIOP‐2001/GPOH) were retrospectively analyzed. These studies included patients from Germany, Austria, and Switzerland between 1993 and 2022. For this analysis only patients from Germany were included as solely for them preventive medical examinations for children were recorded. Four routes to diagnosis were defined:

1. Diagnosis due to tumor‐related symptoms (e.g., painless abdominal swelling, hematuria, etc.^8^),
2. Incidental diagnosis during another illness without tumor‐related symptoms (e.g., viral infection, trauma, headache, etc.),
3. Diagnosis by preventive medical examination^9^ including prenatal diagnosis,
4. Diagnosis by surveillance (abdominal sonography every 3 months up to 7 years of age) in case of a clinically known CPS.

To compare the different routes to diagnosis we furthermore collected data on gender, age, tumor volume at diagnosis and after preoperative chemotherapy, overall and local stage, relapses, and deaths. Details of the whole cohort and for the different routes to diagnosis are given in Table 1.

**TABLE 1:** Characteristics of the patient cohort.

Patients diagnosed						
	All	With tumor‐related symptoms	Incidental diagnosis, without tumor‐related symptoms	By preventive medical examinations	By surveillance for CPSs	Significance
Patient number	2549	1822 (71.5%)	472 (18.5%)	213 (8.4%)	42 (1.6%)	
Gender [%]						
Male/female	45.8 / 54.1	46.2 / 53.8	43.1 / 56.9	52.1 / 47.9	33.3 / 66.7	*p* = 0.057*
Age [years]						
Mean	4.21	4.65	3.78	1.70	2.69	**
Median	3.24	3.57	2.94	1.00	1.83	
STD	9.01	10.39	4.01	1.80	2.62	
Range	0–62.4	0–62.4	0–44.2	0–12.6	0–14.8	
General health status^a^						
Mean	1.74	1.87	1.51	1.21	1.41	*p* < 0.001*
Median	2.0	2.0	1.0	1.0	1.0	
STD	0.82	0.83	0.72	0.48	0.77	
Range	1–5	1–5	1–5	1–4	1–4	
CPS [%]						
Yes	15.2	12.5	17.6	19.7	100	*p* < 0.001*
Localization [%]						
Right	44.8	45.4	43.9	42.7	40.5	*p* = 0.001*
Left	45.7	46.3	46.4	40.4	40.5	
Bilateral	9.5	8.3	9.7	16.9	19.0	
Treatment start with [%]						
Burgery	9.8	7.0	14.8	21.6	16.7	*p* < 0.001*
Preop. CT	90.2	93.0	85.2	78.4	83.3	
Tumor volume at diagnosis [mL]						
Mean	486	549	446	263	119	**
Median	393	460	365	211	21	
STD	417	432	275	240	324	
Range	0.8–4060	0,8–4060	0.9–2027	3.0–1308	1.2–1791	
Missing [*n*]	120	70	26	16	8	
Tumor volume after preop. CT [mL]						
Mean	233	255	180	141	114	**
Median	132	151	93	73	18	
STD	289	304	241	189	241	
Range	0.8–3521	0.9–3521	0.8–1893	1.0–1173	1.4–774	
Missing [*n*]	636	391	139	85	21	
Metastasis [%]						
	17.4	20.9	10.8	5.2	4.8	*p* < 0.001*
Local stage [%]						
I	58.7	54.2	66.1	77.0	78.6	*p* < 0.001*
II	21.6	24.6	16.1	11.7	2.4	
III	18.2	20.3	16.1	7.5	4.8	
Missing	1.5	0.9	1.7	3.8	14.3	
Histology [%]						
Low risk	4.0	4.3	3.2	2.3	7.1	*p* < 0.001*
Intermediate risk	80.4	81,9	78.0	78.4	52.4	
High risk	12.2	12.2	13.3	9.4	14.3	
Nephroblastomatosis	3.4	1.6	5.5	9.9	26.2	
Outcome						
Relapses [%]	12.8	13.2	12.1	9.9	19.0	*p* = 0.304*
5‐year RFS	0.87	0.86	0.88	0.90	0.78	*p* = 0.312***
Deaths [%]	5.4	5.6	5.8	2.8	0.0	*p* = 0.076*
5‐year OS	0.95	0.94	0.95	0.98	1.00	*p* = 0.392***

Abbreviations: preop. CT, preoperative chemotherapy; RFS, relapse free survival; OS, overall survival.

^a^ 1, normal activity; 2, low health impairment; 3, moderate health impairment; 4, severe health impairment; 5, intensive care needed.

* χ^2^ test.

** See figure 2 showing different *p*‐values for different comparisons.

*** Log rank test.

Tumor volume was measured by radiologists using CT or MRI scans according to the ellipsoid formula (volume [mL] = height [mm] × width [mm] × depth [mm] × 0.523). The general health status was defined as 1: normal activity, 2: low health impairment, 3: moderate health impairment, 4: severe health impairment, and 5: intensive care needed.

All data were pseudonymized before statistical analysis and handled according to the general data protection regulation (GDPR) of the EU.

### 2.2 Statistical analysis

Computational and statistical analysis was performed using SPSS 27 for Mac (IBM Corp. Released 2020. IBM SPSS Statistics for Mac 27.0.; 64bit; Armonk, NY, USA). Qualitative and quantitative values are presented as relative and absolute frequencies as well as mean and standard deviation. *t*‐Test for two independent samples was used to compare means between two independent groups. Values not showing a normal distribution were compared using Mann–Whitney *U*‐tests for two independent groups. With the help of *χ* ^2^‐test, Fischer exact test and univariate analysis of a linear model we compared relative frequencies between independent groups. Survival was evaluated by Kaplan–Meier analyses. Cox regression analysis for relapse‐free and overall survival using known risk factors (age, metastasis, tumor volume after preoperative chemotherapy, and histology) together with route to diagnosis were performed. Two‐sided significance was defined as *p* < 0.05 for all statistical tests. We did not account for the issue of multiple statistical testing due to the explorative nature of the investigation. Thus, we report raw, two‐sided *p*‐values.

### 2.3 Ethics statement

For taking part in the clinical trial informed consent was given by the parents or the legal guardians in all cases and in addition by children older than 8 years providing them with age depending information. Missing informed consent was the only reason for not participating in the analysis. All clinical trials were reviewed and approved by the Ethics Committee of the “Ärztekammer des Saarlandes” (/LS of 23/04/1993, no. 136/01 of 20/09/2002 and 248/13 of 13/01/2014).

## 3 RESULTS

### 3.1 Patient population and routes to diagnosis

Between 1993 and 2020, 3258 patients with kidney tumors were registered in the SIOP 93‐01/GPOH and 2001/GPOH trials and studies. In 260 (7.9%) of these patients the route to diagnosis was unknown, resulting in 2998 patients eligible for analysis. Of these, 449 (14.9%) patients were registered with a non‐WT.

**FIGURE 1:**
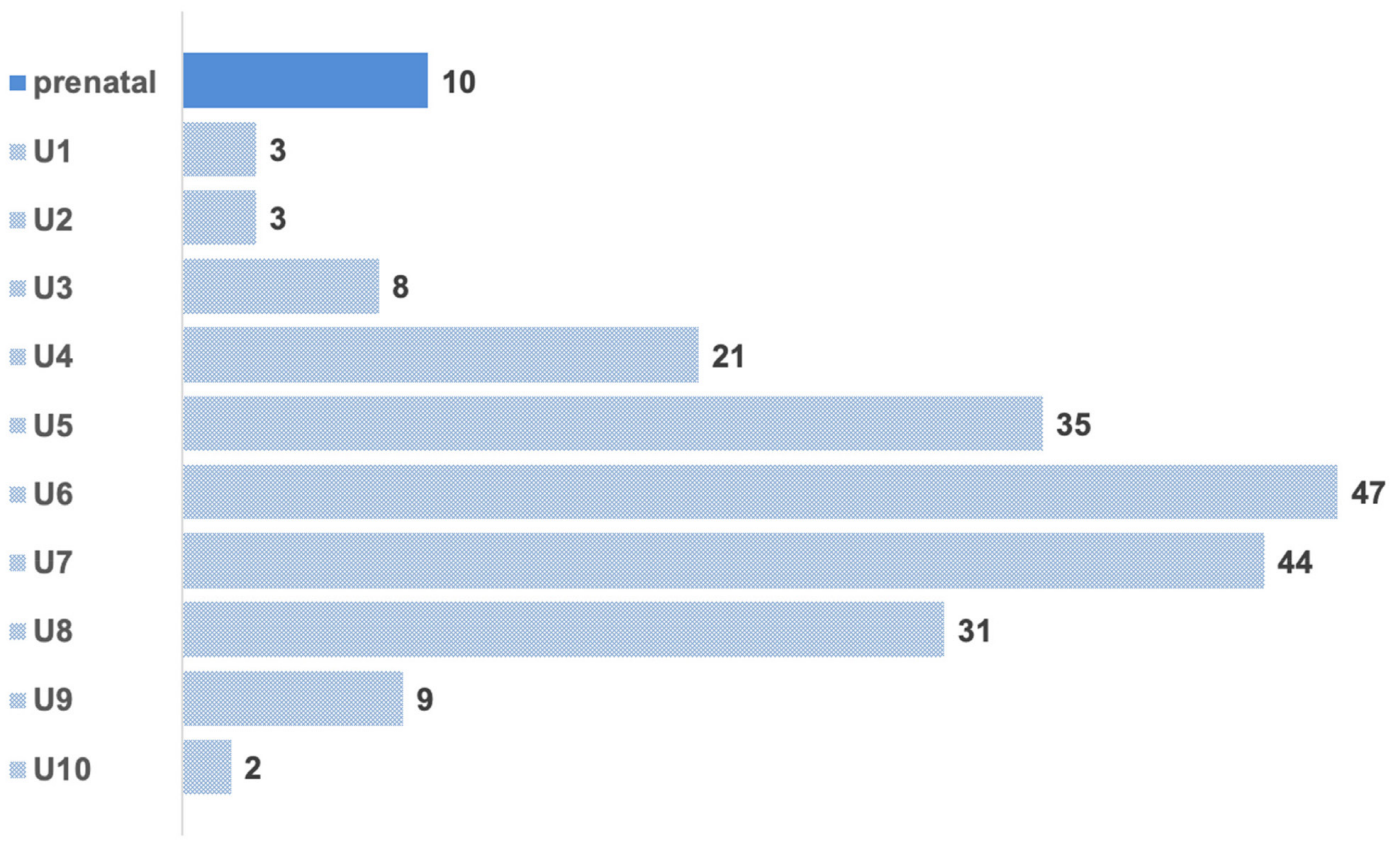
The number of WTs diagnosed prenatally or during preventive medical examinations according to the different scheduled time periods during childhood (U1: directly after birth, U2: 3–10 days of age, U3: 4–6 weeks of age, U4: 3–4 months of age, U5: 6–7 months of age, U6: 10–12 months of age, U7: 21–24 months of age, U8: 3.5–4 years of age, U9: 5–5.25 years of age, U10: 13–14 years of age).

Of the remaining 2549 patients 1822 (71.5%) were diagnosed with tumor‐related symptoms, 472 (18.5%) incidentally during another disease without tumor‐related symptoms, 213 (8.4%) by preventive medical examinations (Figure 1) or prenatal diagnosis (10; 0.4%) and 42 (1.6%) by surveillance in case of a CPS (Table 1). In the latter group, 16 patients had Beckwith‐Wiedemann syndrome (BWS), nine isolated hemihypertrophy, 13 WAGR (WT, aniridia, urogenital malformations and range of developmental delays), four Denys–Drash syndrome (DDS), and four patients genitourinary malformation that did not fulfill the clinical criteria for WAGR or DDS. Comparing the number of patients with WT enrolled in this analysis with the number of patients with WT from the comprehensive German Childhood Cancer Registry, 99.4% were enrolled in the clinical trials between 2008 and 2022 (https://www.kinderkrebsregister.de/, accessed on December 2, 2023), making our study cohort representative for all patients with WT [12].

### 3.2 Analysis of gender, age, general health status, tumor volume, metastasis, and stage distribution

Only for gender no significant differences between the distinct routes to diagnosis were found.

**FIGURE 2:**
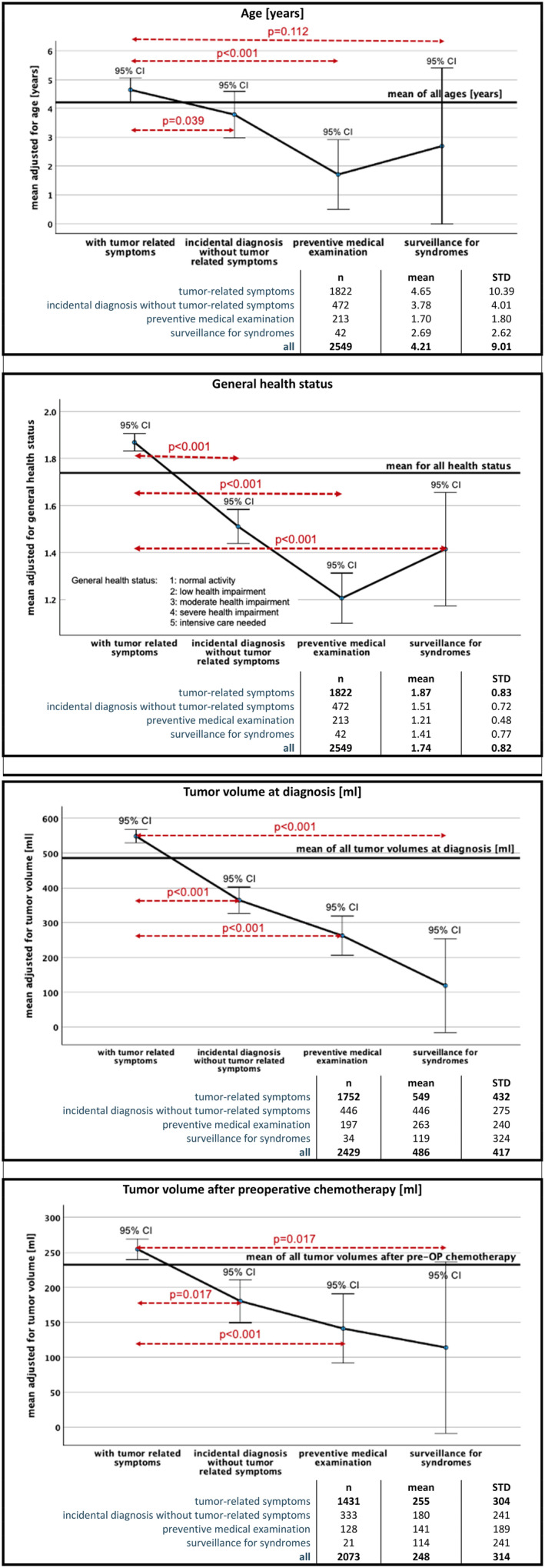
Patient and tumor characteristics depending on the route to diagnosis. CI, confidence interval.

The median age of patients varied significantly from 4.65 years in children diagnosed with tumor‐related symptoms to 1.70 years in those diagnosed by preventive medical examination (*p* < 0.001). Interestingly, children diagnosed by surveillance for a CPS were not the youngest in this cohort (2.69 years) (Figure 2). Furthermore, general health status also significantly differed (*p* < 0.001) between these groups (Table 1, Figure 2). As children with a CPS may also encounter health problems related to the CPS, the health status of these children was worse despite their smaller tumor volume compared to those detected by preventive medical examinations (Figure 2). The mean and median tumor volume at diagnosis and after preoperative chemotherapy was the highest for children having tumor‐related symptoms ranging between 119 mL and 549 mL at diagnosis and 114–265 mL after preoperative chemotherapy (*p* < 0.05; Figure 2). Reduction of tumor volume after preoperative chemotherapy was between 52% and 62%, except for children diagnosed by surveillance with nearly no shrinkage (4%). In this group of patients significantly more bilateral diseases and nephroblastomatosis was found (*p* = 0.001; Table 1). According to tumor volume the percentage of metastasis was highest in patients with tumor‐related symptoms (20.9%) compared to 4.8% if WT was found by surveillance (*p* < 0.001). In the latter group, the local stage distribution after surgery was also the best compared to all other routes to diagnosis with 78.6% stage I and only 4.8% stage III tumors (*p* = 0.008 Table 2).

**TABLE 2:** Cox regression for 5‐year relapse free and overall survival. Categorial variables with more than two categories are tested in case of route to diagnosis: with tumor‐related symptoms against all others; in case of local stage: stage I against all others and in case of histology: low risk against all others.

	Hazard ratio	95% confidence interval for Hazard ratio	*p*‐value
*5‐year relapse free survival*			
Route to diagnosis (with tumor symptoms)			0.997
Incidental, without tumor‐related symptoms	1.059	0.776–1.575	0.821
By preventive medical examination	1.002	0.524–1.812	0.997
By surveillance in case of CPSs	0.000	0.000–>100	0.402
Age	1.055	1.002–1.111	**0.043**
TV after preop CT	1.000	1.000–1.001	0.225
Local stage (stage I)			**0.003**
Stage II	1.645	0.965–2.805	0.068
Stage III	2.323	1.425–3.785	**< 0.001**
Metastasis	2.480	1.633–3.767	**< 0.001**
Histology (low risk)			**< 0.01**
Intermediate risk	2.459	0.596–10.142	0.213
High risk	8.743	2.070–36.929	**0.003**
Nephroblastomatosis	0.000	0.000	0.987
*5‐year overall survival*			
Route to diagnosis (with tumor symptoms)			0.952
Incidental, without tumor‐related symptoms	1.145	0.658–1.992	0.632
By preventive medical examination	0.848	0.253–2.834	0.788
By surveillance in case of CPSs	0.000	0.000 o >100	0.971
Age	1.029	0.966–1.097	0.373
TV after preop CT	1.000	1.000–1.000	0.339
Local stage (stage I)			**0.008**
Stage II	2.474	1.337–4.579	**0.004**
Stage III	2.120	1.168–3.848	**0.013**
Metastasis	3.585	2.215–5.804	**< 0.001**
Histology (low risk)			**< 0.001**
Intermediate risk	1.227	0.292–5.144	0.780
High risk	7.457	1.759–31.623	**0.006**
Nephroblastomatosis	0.000	0.000	0.985

*Note*: Bold values are only given for significant *p* values.

### 3.3 Analysis of outcome

**FIGURE 3:**
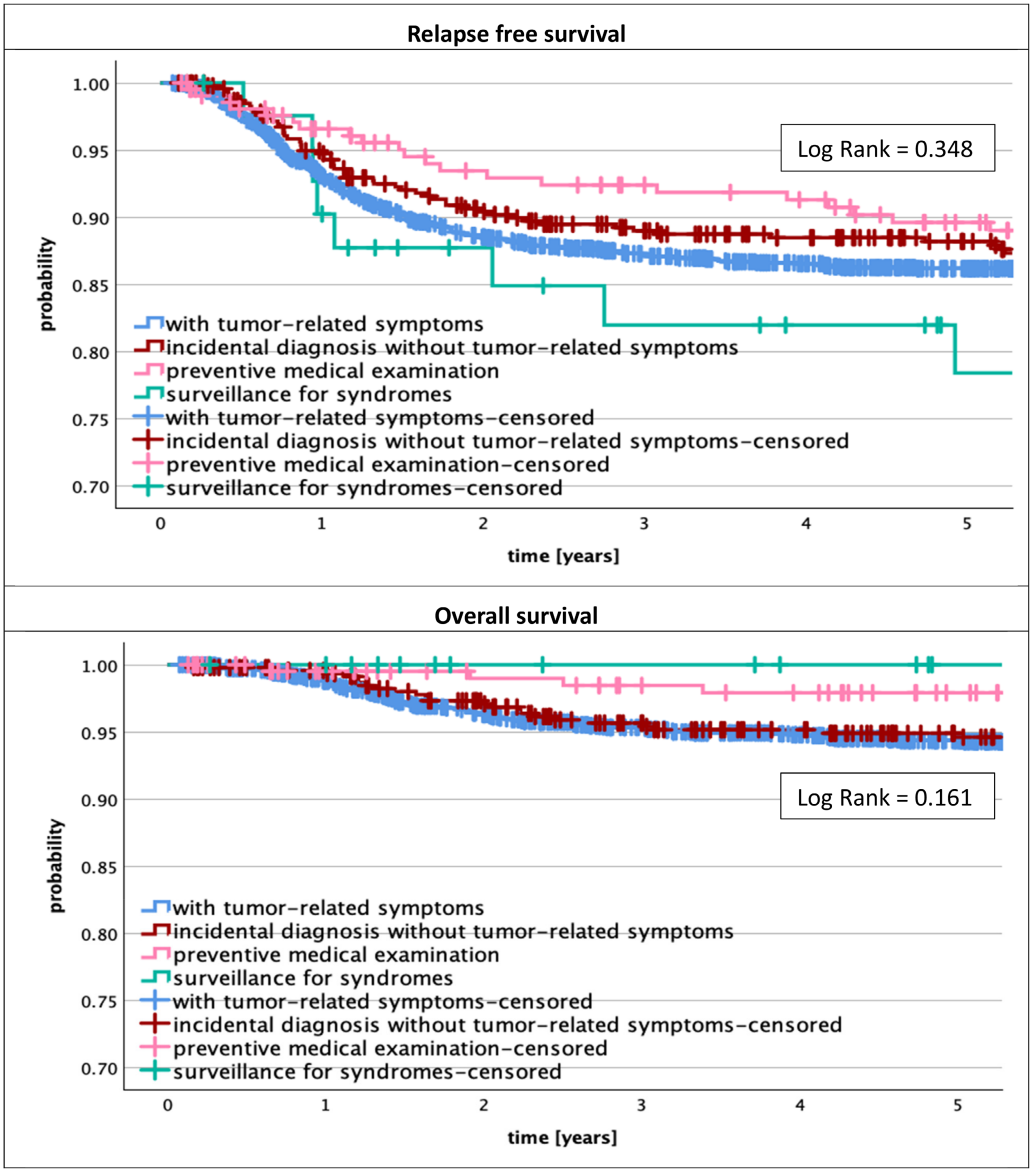
Relapse free and overall survival depending on route to diagnosis.

The outcome of patients defined by relapse and death is not significantly different between the different routes to diagnosis. The highest percentage of relapses was found in patients diagnosed by surveillance (19.0%), but their relapses could be cured and none of them died within 5 years of diagnosis. This is different for all other routes to diagnosis in whom not all patients could be rescued. Life tables according to Kaplan–Meier for relapse free (RFS) and overall survival (OS) are given in Figure 3. There is no significant difference found in RFS and OS between the different routes to diagnosis. In addition, the Cox regression (Table 2) shows significant differences for RFS and OS only for local stage (*p* = 0.003 (RFS), *p* = 0.008 (OS)), metastasis (*p* < 0.001 (RFS and OS)), and histology (*p* < 0.01 (RFS), *p* < 0.001 (OS)), which are the most relevant clinical risk factors for WT, whereas the route to diagnosis does not play a role for RFS and OS in this analysis. For RFS age was identified as an additional risk factor in the Cox regression analysis (*p* = 0.043).

## 4 DISCUSSION

The main focus of this paper is to show the impact of different routes to diagnosis on tumor burden as a hint to time of diagnosis and outcome. It is well acknowledged that early diagnosis and treatment of cancer increases the chances of a cure and reduces the risk of toxic side effects.^10^, ^11^ This in turn leads to an improved quality of life for cancer survivors.^12^ The route to diagnosis of cancer depends on interactions between a patient and the healthcare system that is responsible for an early diagnosis. Linking Cancer Waiting Times data, data from cancer screening programs and cancer registration data may help to understand and explore delays in cancer diagnosis.^13^ A delay in cancer diagnosis and start of treatment can be divided into three phases: the first one from initial symptoms to first medical contact, the second one between the first contact and cancer diagnosis and the third from diagnosis to the start of treatment.^14^, ^15^, ^16^ In childhood cancer, Dang‐Tan and Franco et al. found that the delay caused by physicians was longer than the delay caused by patients.^12^ The median delay ranged from 2.5 weeks for WT to 29.3 weeks for brain tumors. During the Covid pandemic there was a higher proportion of advanced disease and larger tumor volumes in WT, but the time to the start of treatment was not delayed.^17^ In older patients (adolescents and young adults) with WT a higher tumor burden can be found^18^ which may be caused by a longer time to diagnosis also resulting in a poorer outcome.

### 4.1 The impact of different healthcare systems

Our analysis did not address the delay in diagnosis, as the time spans between first symptoms, visiting a physician, diagnosis of WT, and start of treatment were not recorded in our database. We focused on four routes to diagnosis, including symptomatic patients, those with incidental diagnosis due to other diseases, or during a preventive medical examination or screening programs for CPSs. We did not separate emergency presentation as an additional subgroup like Ruddy et al.^19^ Instead, these patients were included in the group of those with tumor‐related symptoms without further specification. We analyzed if the route to diagnosis correlates with the age of the child at diagnosis, general health status at diagnosis, tumor volume, tumor stage, and outcome. In adult patients with urological cancers, the outcome is strongly linked to the route to diagnosis (Douglas‐Moore et al. 2017). In England, a “routes to diagnosis” project,^13^ not specifically addressed to childhood cancer, categorized the pathways that lead to a patient's diagnosis and provided valuable information for monitoring system changes in cancer pathways and improving patient outcomes. The project was set‐up due to the lower cancer survival in England compared to other parts of Europe, which was also shown for WT before.^20^ In an analysis by Pritchard‐Jones et al., a delay in diagnosis for WT in the UK could be found by comparing tumor volume and overall staging in WT to Germany with higher local stage I (55.1% vs. 35.6%) and less metastatic disease at diagnosis (15.6% vs. 18%) in Germany.^21^ This disparity could be explained by different health care systems since children in the UK primarily have to visit a general practitioner (GP), whereas in Germany a pediatrician is usually the first contact point to the health care system after birth. Besides well‐trained physician examination skills, a guide for early recognition of childhood cancer based on signs and symptoms is essential for primary care physicians to avoid missing early diagnosis and to be aware of highly suspicious symptoms (Fragkandrea, Nixon, and Panagopoulou 2013).

In contrast to adults, Childhood cancers are generally progressing rapidly. In WT the doubling time of the tumor volume is around 2–3 weeks as exemplified by a patient with WT whose parents refused treatment for several weeks.^22^ Larger tumors are also seen in Sub‐Saharan Africa, where WT diagnosis is delayed due to many other factors.^23^ In a retrospective study from a single center in India, age, the education of the mother and the cancer type were found to be the most important factors predicting the time to diagnosis and treatment.^24^ In summary, in low and middle income countries, several diagnostic barriers can impair health care in general^1^ leading to later diagnosis.

### 4.2 Diagnosis within a surveillance program

In adult oncology, Alelyani et al. reviewed the routes to diagnosis in adult cancer patients and found that self‐examination and screening procedures are only helpful in cancers that are visible, whereas others are discovered after disease progression due to symptoms.^25^ Screening programs as in adults are not useful in children as most of their cancers are not visible at early stages. Therefore, screening for WT is only done in patients with CPSs. In our cohort, only 1.6% of patients with WT were diagnosed through screening. Interestingly, a much larger proportion of patients in our cohort had a CPS (15.2%), but most of them were not enrolled in an abdominal ultrasound screening program (13.6%). These CPSs remained undetected until the diagnosis of WT. This can be explained by the distinct signs of CPSs that become more pronounced with increasing age.^26^ In these patients, the general health status is worse than in those diagnosed during preventive medical examinations, likely due to health issues caused by the underlying CPS. Interestingly for the whole group of these patients there is no tumor volume change after preoperative chemotherapy. This may be due to genetics, for example, WT1 mutations linked to stromal type and TRIM28 inactivation to epithelial type as both histological types do not shrink under preoperative chemotherapy. In our cohort of patients with WAGR and Denys–Drash syndrome the median volume at diagnosis was 228 mL and after preoperative chemotherapy even 277 mL. On the other hand, the tumors of patients with BWS shrinked from 123 to 36 mL after preoperative chemotherapy.

### 4.3 Diagnosis without tumor‐related symptoms

Besides diagnoses resulting from a screening program, there are two other groups of patients in our cohort in whom WT was incidentally detected without tumor‐related symptoms (8.4% during a preventive medical examination and 18.5% in case of another disease). Similar numbers diagnosed by preventive medical examinations were described first by Gutjahr et al.^8^ Altogether 28.5% of the WT patients in our cohort were diagnosed without tumor‐related symptoms. These patients were diagnosed earlier, had better general health conditions, their tumor volumes were smaller and local and overall stage was favorable with less metastasis at diagnosis and a higher percentage of local stage I after surgery.

### 4.4 Outcome analysis

As treatment in WT is very efficient, there was no significant difference in RFS depending on tumor‐related symptoms. Interestingly, patients diagnosed after screening had a tendency towards lower RFS, which can be explained by more patients with bilateral diseases and nephroblastomatosis and the development of metachronous tumors because of their CPS^7^. Nevertheless, none of these relapsed patients died, resulting in a 5‐year OS of 100%. This underlines the importance of regular sonographic screening in this particular patient group with regard to early diagnosis associated with smaller tumor volumes. Even if there is no difference in outcome, it is important to note that the patients without tumor‐related symptoms received less intensive treatment due to their lower overall and local stage. Especially anthracyclines and radiotherapy could be avoided in many of them. This will result in less late effects and better quality of life, which underlines that an early diagnosis of WT, characterized by missing tumor‐related symptoms, is relevant. Welter et al. also discussed the importance of early diagnosis in CPS associated with WT^27^ being in accordance with our findings that patients with tumor‐related symptoms have larger tumor volumes and more metastatic disease. In addition, Qian et al.^18^ could show that older age in patients diagnosed with WT is independent of other factors associated with worse OS, which further emphasizes the need to ensure early diagnosis, In osteosarcoma, the prognostic impact of diagnostic and treatment delay resulting in higher tumor load was analyzed by Vasquez et al. finding that latency to diagnosis did not influence EFS and OS, but outcome was poorer in patients with a longer time to complete treatment.^28^ This time‐period as a measure for treatment intensity was not analyzed in our study.

### 4.5 Perspective

To our knowledge, there are currently no other studies available that discuss the four routes to diagnosis with respect to gender, age, general health condition, tumor volume, overall and local stage and outcome in WT. In the ongoing SIOP‐RTSG UMBRELLA study, data related to these routes of diagnosis will be prospectively collected. These can be compared between countries and related to the different health care systems in the participating countries in Europe and beyond. This may help to improve early diagnosis also in countries, where preventive medical examinations for children done by pediatricians are rare or not provided. A Danish population‐based study on the recognition of early signs of childhood cancer and the interpretation of symptoms resulted in a faster diagnosis.^29^ In a review by Starke et al. advice is given on how to start the diagnostic workup based on presenting features and epidemiological data.^10^ Therefore, it is important to educate physicians not only about CPSs and their distinctive signs, but also about when to consider one as a possible diagnosis in a child.^30^ This will enable more patients to benefit from an early diagnosis.

## 5 CONCLUSION

The route to diagnosis of WT is correlated with age at diagnosis, general health status, tumor volume, and stage distribution, but does not significantly impact patient outcomes. Nevertheless, our study underlines the importance of predisposition surveillance programs as well as preventive medical examinations for children allowing earlier diagnosis and thus decreasing toxic treatments resulting in the less late effects and improved quality of live.

## AUTHOR CONTRIBUTIONS

**Marvin Mergen:** Conceptualization (lead); data curation (equal); formal analysis (equal); methodology (equal); validation (equal); visualization (lead); writing – original draft (equal); writing – review and editing (lead). **Nils Welter:** Writing – review and editing (equal). **Rhoikos Furtwängler:** Data curation (equal); formal analysis (equal); investigation (equal); writing – review and editing (equal). **Patrick Melchior:** Writing – review and editing (equal). **Christian Vokuhl:** Writing – review and editing (equal). **Manfred Gessler:** Writing – review and editing (equal). **Clemens‐Magnus Meier:** Writing – review and editing (equal). **Leo Kager:** Writing – review and editing (equal). **Jens‐Peter Schenk:** Writing – review and editing (equal). **Norbert Graf:** Conceptualization (supporting); data curation (equal); formal analysis (equal); funding acquisition (lead); investigation (equal); methodology (equal); project administration (lead); supervision (lead); validation (equal); visualization (supporting); writing – original draft (equal); writing – review and editing (equal).

## FUNDING INFORMATION

This work was partly funded by the German Cancer Aid (Deutsche Krebshilfe, Grant No: 70‐1899 and 50‐2709‐GR2) and the Elterninitiative krebskranker Kinder im Saarland e.V.

## CONFLICT OF INTEREST STATEMENT

The authors declare that they have no conflict of interest.

## CONSENT

Informed consent was obtained from all patients, their parents, or legal guardians involved in the study.

## Data Availability

The data presented in this study are available on request from the corresponding author. The data are not publicly available due to ongoing analysis.
